# Do the Loss of Thigh Muscle Strength and Tibial Malrotation Cause Anterior Knee Pain after Tibia Intramedullary Nailing?

**DOI:** 10.1155/2019/3072105

**Published:** 2019-03-27

**Authors:** Emre Anıl Özbek, Mahmut Kalem, Hakan Kınık

**Affiliations:** ^1^Yozgat City Hospital, Orthopedics and Traumatology Department, Yozgat City Hospital, 66100 Viyana Avenue, Yozgat, Turkey; ^2^İbn'i Sina Training and Research Hospital, University of Ankara, Orthopedics and Traumatology Department, İbn'i Sina Hospital, Ankara University Medicine Faculty, 06100 Samanpazarı, Ankara, Turkey

## Abstract

**Purpose:**

Anterior knee pain (AKP) is a common complication after tibia intramedullary nailing surgery, but yet the etiology is not fully revealed. Our study had two hypotheses. The first one is “after tibia intramedullary nailing with transtendinous approach, thigh muscles strength decreases and this loss of muscle strength causes AKP.” Secondly, “lower extremity rotational profile is affected after tibia intramedullary nailing.”* Methods*. Our study was planned retrospectively and included 40 patients, who underwent tibia intramedullary nailing surgery. Mean follow-up time was 22.5 months. Tegner Lysholm knee scoring scale was applied to evaluate postoperative functional outcomes of all patients. Isometric muscle strengths of bilateral knee extensor and flexor muscle groups were compared with hand-held dynamometer. In addition, bilateral lower extremity Staheli rotational profile angles (foot progression angle (FPA), thigh-foot angle (TFA), and transmalleolar angle (TMA)) were compared.

**Results:**

Lysholm knee score was evaluated as excellent in 28 patients. AKP were detected in 15 patients and there was no significant difference between the injured limb with contralateral quadriceps mean muscle strength (injured limb mean (ILM) = 201.97 Newton (N) – contralateral mean (CM) = 205.4 N). However, there was a significant difference (p<0,05) between injured limb with contralateral extremity hamstring mean muscle strength (ILM = 153.2 N– CM=158.95 N). Although there was a significant difference between the two extremities' rotational profile angles, there was no significant correlation between the rotational profile angles and knee pain.

**Conclusion:**

As a result of our study, AKP appears to be significantly related to the loss of hamstring muscle strength. We suppose that hamstring exercises will gain importance in rehabilitation programs of tibia intramedullary nailing surgery in future.

## 1. Introduction

Tibia fractures are the most common long bone fractures and the surgical treatment varies among surgeons [1]. A large-scale, 5-continent study by Bhandari et al. included 577 orthopedic surgeons and compared their treatment methods for tibia fractures in their daily practices. Nearly all the surgeons in the study indicated that they used the intramedullary nailing (IMN) method for the treatment of tibial fractures; however, it was demonstrated that the surgical approaches they applied during the operations were different. The study reported that surgeons in South America, Asia, Europe, and Australia used the transtendinous approach 3 to 50 times more frequently compared to the parapatellar approach during the IMN surgery, but anterior knee pain (AKP), emerging as a postoperative complication after both surgical approaches at frequencies up to 50%, was reported to be a challenging issue [1]. Although the etiology of this frequently seen complication has not been clarified in the literature yet, some etiologic factors have been suggested including the transtendinous approach, alignment disorders, weakness of the thigh muscles, and heterotopic ossifications [2–4].

Keating et al. reported higher frequencies of pain with the patellar tendon split compared to the parapatellar approach [5]. A study by Weil et al. reported that the AKP occurred less frequently with the lateral parapatellar approach, without traumatization of the infrapatellar fat tissue [6]. However, there are studies in the literature reporting no significant association between surgical approaches and the AKP [2, 3].

The number of studies investigating the association between the AKP and thigh muscle strength, following tibial IMN surgery, is quite rare in the literature. A study conducted by Väistö et al., investigating reamed and interlocking IMN surgery in a group of 28 patients with tibial shaft fractures, reported that the AKP emerged due to the weakness of the quadriceps muscle rather than the hamstring muscle and it regressed in a range from 3 to 8 years by means of appropriate physical therapy and rehabilitation [7]. Another study by Väistö et al. reported that the etiology of postoperative AKP was multifactorial, and the tibia alignment disorder was one of the etiologic factors [4]. Nevertheless, there are no studies in the literature investigating the association between the lower extremity rotational profile alterations and AKP after tibial IMN.

Including patients who underwent tibial IMN by the transtendinous approach, the aim of our study is to examine the association of the postoperative AKP with two factors, which are the thigh muscle weakness and alterations in the Staheli lower extremity rotational profiles. No studies with this design have been found in the literature.

## 2. Material and Methods

Approval from the ethical committee of Ankara University Medical Faculty was obtained for our study. Between 2012 and 2015, 274 tibia intramedullary nailing surgeries were performed in our clinic by the same senior surgeon. Within these patients, 44 patients' surgeries were performed by transpatellar approach and complete and uncomplicated bone healing was observed in 40 patients who were included our study. No patient needed revision surgery and fully bone union was observed of all patients' radiography. Demographic information, the mechanisms of injury, and the preoperative and postoperative radiographs were recorded.

All patients included in the study were given the Tegner Lysholm Knee Scoring Scale (TCSS). The scale has long been established and widely recognized in the worldwide literature as reliable and being practical to be administered [8]. In the TCSS, <65 points were rated as “poor,” 65-83 points as “fair,” 84-90 points as “good,” and> 90 points as “excellent” [2, 7].

**Figure 1:**
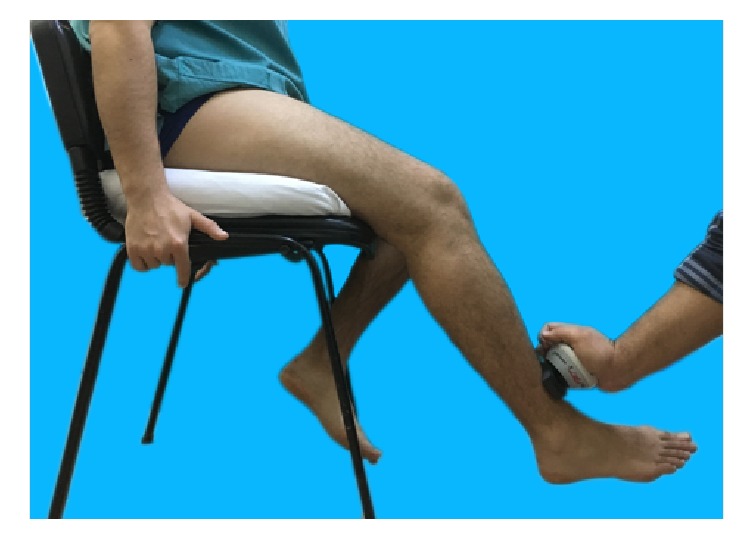
Evaluation of the knee extensor muscles.

**Figure 2:**
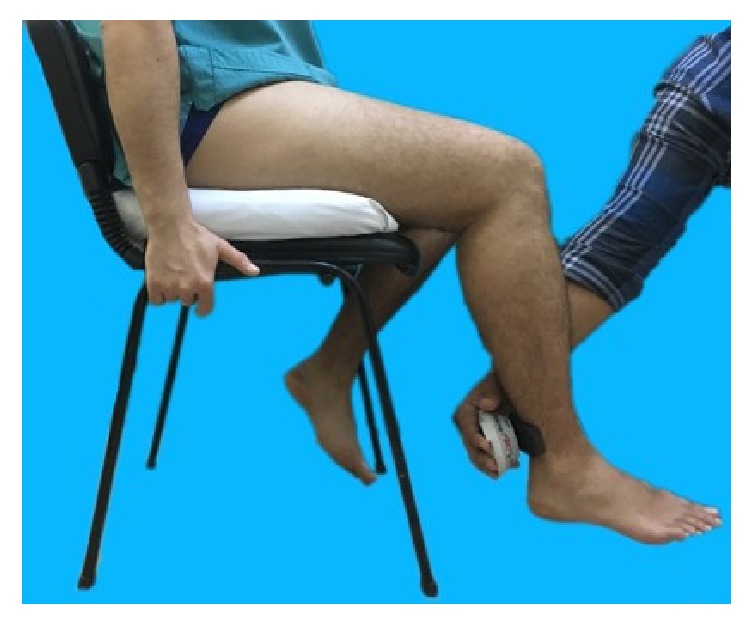
Evaluation of the knee flexor muscles.

Quadriceps and hamstring isometric muscle strengths were measured by means of a digital handheld dynamometer, MicroFet 2 (Hoggan Health Industries, Draper, UT), which was reported to be used reliably in many trials. The muscle strength was recorded in Newtons (N) [9–11] (Figures 1 and 2). Providing verbal encouragements to use their maximum efforts, the patients were asked to apply forces to the dynamometer during a period of 5 to 10 seconds while patients were sitting in a fixed chair also examiner's hand pressed down the thigh to chair. This process was repeated 3 times with 2-minute resting intervals in between. The highest measured value was recorded [9–11].

In the postoperative period, Staheli's rotational profile angles (thigh-foot angle (TFA), transmalleolar angle (TMA), and foot progression angle (FPA)) were measured in both lower extremities as described previously in the literature and were recorded [12].

Multiple statistical methods were carried out during the statistical analysis phase of our study. Descriptive statistical methods were used in our study for classifications and qualification-based combinations. Total, mean, and percentage calculations were performed by the descriptive statistics. The paired t-test was used to test two independent groups. The Wilcoxon test was used to analyze the magnitude of the differences between the paired groups. The Chi-square test was used as a statistical analysis method to compare the data using two-way or more cross tabulations.

## 3. Results

The mean age of the study patients was 44.4 (19-70). Of these patients, 75% of them were males. 35% (n=14) of the patients had open fractures. The distribution of these patients by the Gustilo-Anderson classification was determined to be as follows: 17.5% (n=7) type 1, 7.5% (n=3) type 2 open fracture, and 10% (n=4) type 3 open fracture [13]. The mechanisms of injury detected in our patients were as follows: falls 47.5% (F), gunshot wounds 7.5% (GSW), out-of-vehicle traffic accidents 32.5% (OVTA), in-vehicle traffic accidents 10% (IVTA), and fall from high 2.5% (FH), Table 1.

**Figure 3:**
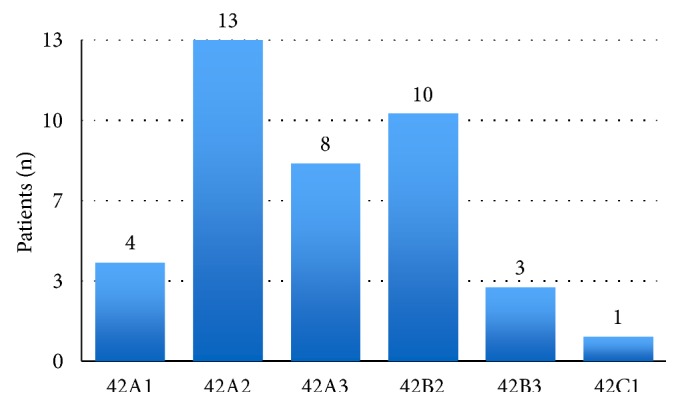
AO classification.

The mean follow-up duration of the study patients was 27.08 months (15-50 months). The preoperative radiograms of the study patients were classified in accordance with the AO classification and their distribution has been presented in Figure 3 [14].

The following parameters were evaluated in the postoperative radiograms of the study patients, including the distance of the nail tip to the tibial plateau “nail presence,” the distance of the nail tip to the anterior cortex of tibia “nail prominence,” and the sum of these two values “nail-apex distance.” In our study, protrusions of the nail tips from the tibial plateau were detected in none of the patients; however, nail tips' protrusions out of the anterior cortex (nail prominence with a negative value) were detected with a mean length of 3mm in 12.5% (n=5) patients. 15% (n=6) of the patients were detected with a nail apex distance longer than 2.5cm (Table 2).

**Figure 4:**
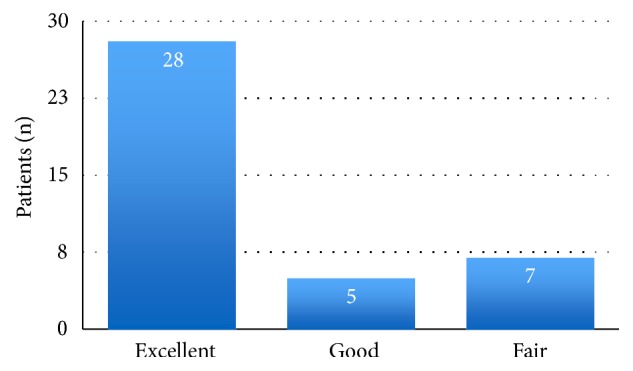
Lysholm knee scores.

The TCSS was administered to all the study patients. The mean score of the patients was 95 (Figure 4).

The isometric strengths of the quadriceps and hamstring muscles were measured in the tibia IMN applied extremities and the contralateral extremities of all study patients. No differences were determined between the affected extremities (Q) and the contralateral extremities (Q Control) in the quadriceps muscle strength (p=0,228). The mean value for the affected extremity was 201.97 (±55.6) N and the mean value for the contralateral extremity was 205.4 (±50.1) N. A significant difference was determined between the affected and the contralateral extremities in the hamstring muscle strength (p<0,001). The mean value for the affected extremity was 153.2 (±34.6) N and the mean value for the contralateral extremity was 158.95 (±47.5) N (Table 3). Also, affected and contralateral extremities' Q and H muscles strengths were compared based on fracture etiologies which were high energy trauma and low energy trauma. However, there was no statically significant differences found between the two groups (p>0.05).

FPA, TFA, and TMA measurements were performed in the affected and contralateral extremities in all study patients. A significant difference was observed between the two extremities in FPA measurement outcomes (p=0,025). The median FPA value of the affected extremity was 19° (1°-30°) and it was 18° (8°-31°) in the contralateral extremity. 8 (25%) patients were detected with differences “>10°” between the two extremities in the FPA values. No significant differences were observed between the two extremities in the TFA measurement outcomes (p=0,394). The median TFA value of the affected extremity was 13° (2°-19°) and it was 12° (1°-20°) in the contralateral extremity. 6 (15%) patients were noted with differences “>10°” between the two extremities in the TFA values. A difference of “>10°” between the two extremities in both the FPA and TFA measurement outcomes was detected in 5 patients (12.5%). A significant difference was found between the two extremities in TMA measurement outcomes (p<0,001). The median TMA value of the affected extremity was 15° (8°-23°) and it was 18° (9°-27°) in the contralateral extremity. 3 (7.5%) patients were detected with differences “>10°” between the two extremities in the TMA values (Table 4).

Of the 40 patients, 37.5% (n = 15) of them complained of knee pain and this pain was thought as AKP which is the postoperative complication of tibia IMN surgery. In this group of patients with the AKP, no differences were observed in the quadriceps muscle strengths of the two extremities, rotational profile angles (FPA, TFA, and TMA), nail apex distances >2.5 cm, and negatively valued nail prominence distances (p>0.05). However, a significant difference was detected between the two lower extremities in terms of the hamstring muscle strengths in the patients with the anterior knee pain. However, none of muscle strength and rotational angle have significant difference between AKP and non- AKP group.

## 4. Discussion

Tibia fractures are among the most frequently encountered conditions by the orthopedic surgeons since they are the most common long bone fractures [2]. In recent years, surgeons have preferred IMN method to the conservative treatments or plate osteosynthesis for the treatment of tibia fractures; however, the postoperative pain complication seen at frequencies up to 50% after the IMN methods of the tibia was reported to be a challenging issue [1]. Orfaly et al. compared the paratendinous and transtendinous surgical approaches in the postoperative AKP. Their study reported that the transtendinous approach caused the complication of postoperative AKP and the authors changed their surgical treatment approaches to the paratendinous one [13].

A prospective study conducted by Keating et al reported that the AKP complication was multifactorial; however, they stated that the transtendinous approach was among the major reasons for this complication [5].

Dewitt et al. compared the differences in the contact pressure in the patellofemoral joint after the parapatellar and transtendinous surgical approaches in 8 knee cadavers. They determined higher increases in the pressure on the lateral facet of the patella and on the medial facet of the patella after the parapatellar and transtendinous approaches, respectively. However, they concluded that after the transtendinous approach, increases in the pressure were present on both patellar facets, which cause chondral injuries, leading to the development of AKP more frequently [14].

Unlike the outcomes of the above mentioned studies, there are other studies in the literature demonstrating that there are no differences between these two surgical approaches in terms of developing the complication of AKP. Court-Brown et al. reported that the AKP was not associated with the applied surgical procedures and the pain complication was either regressed or resolved after explantation of the nails [3]. Vaiströ et al. reported that the AKP was not found to be different after either of the surgical approaches and the pain was either regressed or resolved in most of the patients during the long-term follow-up periods [2].

In the current literature, loss of muscle strength was hypothesized in some studies to explain the etiology of the developing pain after the IMN of the tibia. Väistö et al. followed up 40 patients for the postoperative functional outcomes and muscle strengths after the IMN surgery of the tibia. The study reported the etiology of the AKP to be multifactorial; however, it was reported that the pain originated from the loss of muscle strength in the flexor muscles of the knee rather than that in the extensors [4]. The outcomes of our study are parallel to the study findings in the literature.

In our study, the quadriceps and hamstring muscle strengths were compared in the lower extremities with the IMN of the tibia and in the contralateral extremities. This comparison resulted in no statistically significant differences between the two lower extremities in terms of the quadriceps muscle strengths (p=0,228), but a significant difference was found between the two lower extremities in terms of the hamstring muscle strengths (p<0,001). In addition, of the patients, 15 had the complication of postoperative AKP. In this subgroup of patients, the comparison of the muscle strengths resulted in a statistically significant difference only in the hamstring muscle strengths similarly.

In the literature, the proximal prominence of the nail is among the other leading etiological factors of the complication of the AKP [15]. However, there are studies reporting that the nail prominence has no influences on the AKP in the long-term follow-up [15–17]. In our study, the prominence, presence, and nail apex distances of the nail tip were calculated. The comparison of these values has not led to statistically significant results in the group of 15 patients with AKP, parallel to the outcomes of the 14-year long-term follow-up study by Leafivre et al [17].

The postoperative tibial alignment disorder is reported in the literature among the other major leading etiologic factors of the AKP. However, only the coronal and sagittal alignment disorders have been reported in the literature in these types of studies. No studies have been found in the literature comparing the association of the pain with the postoperative rotational alignment disorder in the tibia in patients treated with the IMN method due to tibia fractures. However, there are studies examining the malrotations after the IMN and the effects of these malrotations on the femora-tibial and patella-femoral joint surfaces [18–24]. A study by Kenawey M. et al., examining the rotation deformities and the pressure increases on the tibiofemoral joint surfaces on the lower extremities of 8 cadavers, reported increases in the medial compartment pressure by external rotations and it was reported that this pressure was decreased by internal rotations [24].

In our study, FPA, TFA, and TMA were evaluated in the operated lower extremities in comparison with the contralateral extremities of the patients, who underwent IMN surgery of the tibia. This evaluation resulted in statistically significant differences in the FPA and TMA parameters (p=0,025) (p<0,001). In spite of these statistically significant results, the distribution of the outcomes of these parameters is within the normal distribution limits reported in the literature as “-3° and +20°” for FPA, “-5° and +30°” for TFA, and “0° and +45°” for TMA. The values within these limits are regarded as normal. Therefore, our statically significant results could be assumed rotational differences between lower extremities instead of malrotation. When the patients having a difference of more than 10° between the two extremities were evaluated for the complication of AKP, no statistically significant results were obtained (p>0.05). The outcomes of our study suggest that postoperative malrotations do not lead to the complication of pain. However, none of muscle strength and rotational angle have significant difference between AKP and non- AKP group. The cause of these results could be interpreted as our study contained small sample size of patients with AKP (n=15); therefore no significant difference was found.

The developing anterior knee pain after the intramedullary nailing surgery of the tibia is regarded as a complication with multifactorial etiologies. In our study, investigating almost all the etiologic factors in the literature as regards the anterior knee pain complication developing after the intramedullary nailing surgery of the tibia, the only parameter with a statistically significant correlation to the pain complication, was determined to be the loss of strength in the hamstring muscles. Although the actual cause of AKP is not known in the light of this information, further studies will hypothesize that hamstring exercises will play a wider part in the rehabilitation programs after the tibia intramedullary nailing surgeries in the future.

## Figures and Tables

**Table 1:** Basic characteristic of patients.

Age (years)	44,4 ± 12,95

Sex	

Female	25% (n=10)

Male	75% (n=30)

Fracture Site	

Right	60% (n=24)

Left	40% (n=16)

Mechanism of Injury	

Fall	47,5% (n=19)

MVC	%45 (n=18)

Gunshot Wound	7,5% (n=3)

Hospital Time (days)	3,35 (2 - 7)

Anterior Knee Pain (%)	37,5 % (n=15)

**Table 2:** Basic characteristic of patients.

Gustilo Anderson Class.	

Closed	65% (n=26)

Type 1	17,5% (n=7)

Type 2	10% (n=4)

Type 3	7,5% (n=3)

Nail - Apex Distance (mm)	1,77 ± 0,84

Nail Protrusion (mm)	0,33 ± 0,37

**Table 3:** Thighs muscles strength.

	Mean Value	St. Deviation	p Value
Q Affected Extremity	201,9 7 N	± 55,6	p=0,228
Q Contralateral Extremity	205,4 N	± 50,1	

H Affected Extremity	153,2 N	± 34,6	p<0,001
H Contralateral Extremity	158,9 N	± 47,5	

Q: Quadriceps Muscle, H: Hamstring Muscle

**Table 4:** Staheli rotational profile.

	Median Value	Min - Max	p Value
FPA Affected Extremity	19°	1° - 30°	p=0,025
FPA Contralateral Extremity	18°	8° - 31°	

TFA Affected Extremity	13°	2° - 19°	p=0,394
TFA Contralateral Extremity	12°	1° - 20°	

TMA Affected Extremity	15°	3° - 23°	p<0,001
TMA Contralateral Extremity	18°	9° - 27°	

FPA: Foot Progression Angle, TFA: Thigh-Foot Angle, TMA: Transmalleolar Angle

## Data Availability

The data used to support the findings of this study are available from the corresponding author upon request.
